# Protein-directed synthesis of highly monodispersed, spherical gold nanoparticles and their applications in multidimensional sensing

**DOI:** 10.1038/srep28900

**Published:** 2016-06-29

**Authors:** Yumin Leng, Ling Fu, Liqun Ye, Bo Li, Xiumei Xu, Xiaojing Xing, Junbao He, Yuling Song, Chaoliang Leng, Yongming Guo, Xiaoxu Ji, Zhiwen Lu

**Affiliations:** 1 College of Physics and Electronic Engineering, Nanyang Normal University, Nanyang 473061, China; 2 College of Agricultural Engineering, Nanyang Normal University, Nanyang 473061, China; 3 College of Chemistry and Pharmaceutical Engineering, Nanyang Normal University, Nanyang 473061, China

## Abstract

An *in-situ* reduction method has been reported to prepare gold nanoparticles (GNPs) of 40–110 nm by using the green reducing agents of proteins, which are activated by H_2_O_2_ and the superoxide anion (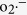). The protein of collagen turns HAuCl_4_ to the aqueous Au(I) ainions, which are further reduced by other proteins to be highly monodispersed and spherical GNPs of different sizes. The GNPs reduced by different proteins are found to be with the exposed {100} facets, the distinctive UV-vis absorption spectra and various colors (See Fig. 1). By means of extracting the color responses, such as red, green and blue (RGB) alterations, an *in-situ* reduction method-based multidimensional sensing platform is fabricated in the process of GNPs synthesis. Without further modification of GNPs, nine common proteins are found to be well detected and discriminated at different concentrations. Moreover, this sensing platform also demonstrates great potentials in qualitative and semiquantitative analysis on the individuals of these proteins with high sensitivity. Furthermore, the validation of this multidimensional sensing platform has been carried out by analysis on the spiked proteins in human urine and the target proteins in complex matrix (e.g. lysozyme in human tear).

Gold-based nanomaterials have attracted much attention in many areas of chemistry, physics, materials science, and biosciences because of their size- and shape-dependent optic, electric, and catalytic properties. Synthesis of spherical gold nanoparticles (GNPs) involves the chemical reduction of chloroauric acid (HAuCl_4_) typically using sodium borohydride (NaBH_4_) and sodium citrate as the reducing agents^1^,^2^, producing particles with sizes of 2–10 and 10–40 nm, respectively. To date, seeded growth strategies are usually used to efficiently synthesize uniform GNPs larger than 40 nm^3^,^4^,^5^,^6^. That is, small GNPs, obtained via NaBH_4_/citrate reduction, are employed as gold seeds and HAuCl_4_ is reduced to Au^0^ atop the seeds by using additional reducing agents, such as ascorbic acid^3^,^4^, hydroxylamine^5^, mercaptosuccinic acid^6^, hydroquinone^7^, and N_2_H_4_·2HCl^8^. However, most of these chemicals are highly active and have potential environmental and biological risks, which might be an issue for their wide applications. The environmental friendly approach to synthesize GNPs by using natural macromolecules has been attracted growing interest in the last few decades^9^,^10^,^11^,^12^,^13^. For example, the groups of Yang used chitosan for reduction of Au^3+^ and stabilization of GNPs^9^. Cellulose has been reported as reducing agent for the “green” synthesis of GNPs^10^. The spider-silk fiber has been utilized as reducing agent to synthesize GNPs^11^. The green natural compounds, plant extracts, were reported to synthesize well-dispersed GNPs^12^,^13^. Among the biomacromolecules, proteins that have been widely used in nanoclusters (<1 nm) synthesis^14^,^15^,^16^ are the ideal candidate for synthesis of GNPs. Herein, we present the first reported an *in-situ* reduction method to synthesize the monodispersed and spherical GNPs with sizes of beyond 40 nm by using proteins, the reducing powers of which are activated by H_2_O_2_ and the superoxide anion (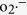). The *in-situ* reduction method to synthesize GNPs as shown in Fig. 2, addition of certain proteins (e.g. collagen (Col)) to HAuCl_4_ solution, turns Au(III) to Au(I) anions, which are further reduced by other proteins in the presence of luminol and H_2_O_2_, to form spherical GNPs with different sizes.

The size-dependent optical properties and good biocompatibility of the prepared GNPs, show us that they have potential in application (e.g. drug delivery, biodiagnostics, optical imaging, detection of ions and biomolecules)^17^,^18^,^19^,^20^. Besides, the green-chemical reducing and stabilizing agents of proteins, which can withstand a wide range of pH conditions due to their complex 3D structures^21^,^22^, make the prepared GNPs being a great application prospect in many areas. Considering that the relative and absolute level of proteins in human body is directly related to specific disease states^23^,^24^,^25^,^26^, we are interested in seeking an efficient method to identify protein balances by using the size-dependent optic properties of GNPs. The prepared GNPs with different sizes and colors are investigated in the field of multidimensional sensing. Recently, multidimensional sensing platforms have been employed for pattern recognition analysis of multiple analytes, especially proteins^27^,^28^,^29^,^30^,^31^,^32^,^33^,^34^,^35^,^36^,^37^,^38^. For example, the GNPs modified by fluorescent polymer or proteins have been applied to detect proteins^27^,^28^. Liu *et al*. presented a multidimensional sensor for pattern recognition of proteins with DNA sequences modified GNPs (DNA−GNPs)^29^. Yan *et al*. reported the simultaneous exploration of the fluorescence, phosphorescence, and scattering properties of manganese-doped ZnS quantum dots as a multidimensional sensing device for the discrimination of proteins^32^. The groups of He and Zhang prepared dual-ligand cofunctionalized gold nanodots and quantum dots to detect proteins via fluorescence response^34^,^35^, respectively. These efforts have made great progress toward developing the multidimensional sensing systems for the discrimination of proteins, however, they either require complicated modification process and/or need a variety of techniques (or instruments) to acquire sensing signals, thus limiting their widespread applications. It is important that such studies achieving the discrimination of proteins in the process of GNPs synthesis, without further modification and fluorescence excitation, are currently unavailable. In this paper, a multidimensional sensing platform is created based on the *in-situ* reduction method to produce the distinct color of GNPs and simultaneously differentiate proteins, simply by extracting the color changes of the Au(I) anions, luminol and H_2_O_2_ mixture in the absence and presence of different proteins. Compared with the reported sensor arrays, three obvious advantages of this sensor array might be particularly attractive: i) Protein can be immediately discriminated in the GNPs synthesis process, without further surface modification; ii) Protein discrimination can be achieved only by extracting color responses, without the utilization of expensive instruments; iii) The as-developed sensing platform also exhibits great potentials in semiquantitative analysis of individual protein with high sensitivity. In final, the as-developed multidimensional sensing platform is validated by analysis of the spiked proteins in human urine and determination of target proteins in complex matrix (e.g. lysozyme in human tear).

## Results

### Synthesis of GNPs by using different proteins

**Figure 3:**
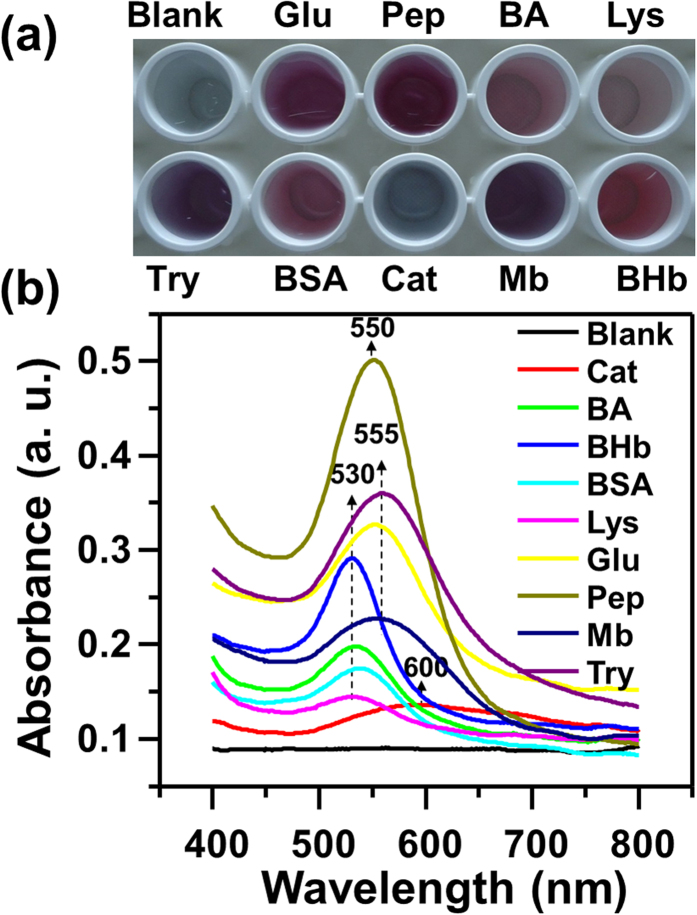
Comparison of (**a**) the colors and (**b**) UV-vis absorption spectra of spherical GNPs reduced by different proteins in the presence of luminol and H_2_O_2_.

Herein, the light-yellow of HAuCl_4_ solution is firstly reduced by Col in strong alkaline solution, to produce the colourless solution, the absorption spectrum of which is shown in Figure S1 (Supplementary Information (SI)). The absorption peak of the colourless solution appears at 246 nm, suggesting that the Au(III) complexes are reduced by Col to aqueous Au(I) anions (e.g. [AuCl_2_]^−^)^39^,^40^,^41^. Inspired by the knowledge that certain proteins (e.g. BSA, Pep and Lys) show the reducing ability to be developed for the preparation of nanoclusters^14^,^15^,^16^, and the reduction potential of Au(I) → Au(0) is lower than that of Au(III) → Au(0)^7^. We postulate that Au(I) is more easier reduced by proteins to produce GNPs. Based on this assumption, we first added the representative proteins (e.g. Lysozyme (Lys), bovine hemoglobin (BHb), pepsin (Pep), trypsin (Try), Bovine serum albumin (BSA), myoglobin (Mb), catalse (Cat), glucoamylase (Glu) and bovine albumin (BA)) to the aqueous Au(I) anions, only Pep and Try turn the colourless of Au(I) to be the red and purple of GNPs (See Figure S2, SI), respectively. Although the results verified the reducing ability of Pep and Try, the relatively lower reducing power of the other proteins failed to turn Au(I) anions to be GNPs. However, keeping in mind that the activities of proteins might be modified with the presence of activators (e.g. H_2_O_2_ and O_2_.^−^)^42^,^43^, H_2_O_2_ and its reaction product of O_2_.^−^ were attempted to activate the reducing power of proteins. The O-O bond of H_2_O_2_ might be broken up and turned into double ^•^OH radicals by the catalytic function of gold^44^. The formed ^•^OH radicals could react with H_2_O_2_ and luminol anion to generate O_2_.^−^ and the luminol radical^31^. The activities of proteins might be also significantly improved in the process of O_2_.^−^ formation^43^. It’s astonishing to us, much stronger reducing power of proteins is observed with the addition of luminol and H_2_O_2_. As shown in Fig. 3a, in the presence of luminol and H_2_O_2_, proteins turn the colourless solution of Au(I) anions to produce the GNPs solution of different colors.

**Figure 4:**
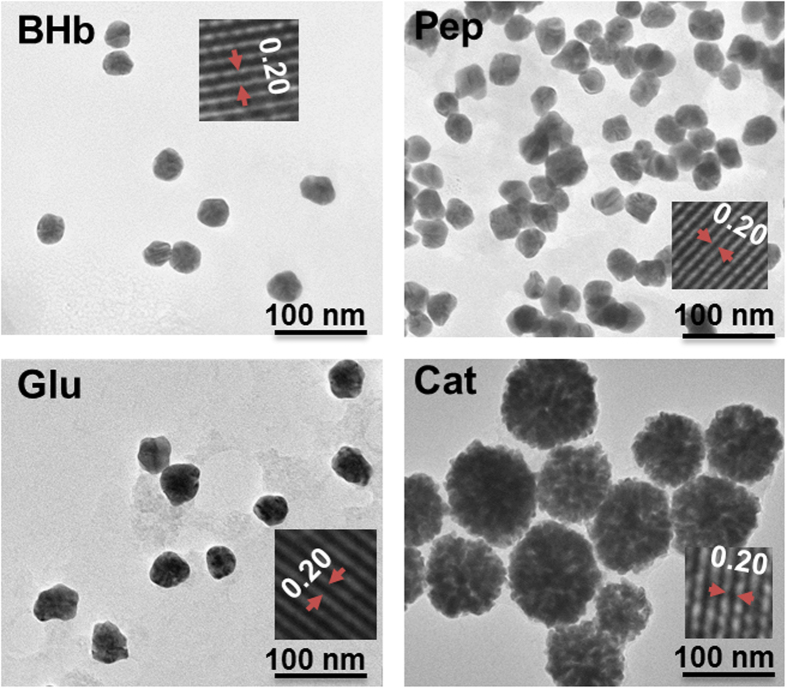
Electron micrographs of spherical GNPs, synthesised through the representative proteins reduction in the presence of luminol and H_2_O_2_. The interplanar distances of all GNPs determined by using ImageJ software are approximately 0.20 nm as shown in the insets.

The properties associated with GNPs can be generally exhibited by UV-vis absorption spectroscopy^1^,^2^,^3^,^4^,^5^,^6^,^7^, which is employed to elucidate the variation of optical properties of GNPs with different sizes. According to Mie theory^45^, the position and intensity of the absorption bands of ultraviolet-visible spectra are strongly influenced by the particle size and concentration of NPs. As shown in Fig. 3b, the UV-vis absorption intensities of as-synthesized GNPs reduced by different proteins are different and the corresponding peaks appear at ~530 nm (Lys, BSA, BA and BHb), 550 nm (Pep), 555 nm (Mb, Glu and Try) and 600 nm (Cat), which mean that the produced GNPs are of different concentrations and sizes. The UV-vis absorption spectra of GNPs are found of characteristic for a given protein. Particle sizes determined from measurement of transmission electron microscopy (TEM) images are beyond 40 nm for all the GNPs reduced by the above-mentioned nine proteins. The highly monodispersed and spherical GNPs reduced by the representative four proteins are shown in Fig. 4. The mean diameters of GNPs reduced by the representative proteins of BHb, Pep, Glu and Cat, are determined to be 43.56 nm, 44.21 nm, 45.23 nm and 102.25 nm, respectively. Interestingly, the denser GNPs reduced by BHb, Pep and Glu can be observed, while the GNPs reduced by Cat seem to be flower-like clusters. The reason might be that the assembly kinetics of Cat is reaction limited and the structure of Cat is insufficiently dense to assemble Au(0). Due to the unique structural and mechanical functions of proteins, the GNPs of different shapes and diameters are formed in the protein-directed synthesis process. Moreover, the various functional groups (e.g. –OH, –NH_2_, –SH and –COOH) of different proteins could impact on the size and shape of synthesized GNPs. For example, the number and position of thiol group (–SH) in proteins are essential in the synthesis of GNPs^46^. The functional groups induce proteins to adsorb on the GNPs surface via Au-O, Au-N, Au-S and Au-C interactions, their stretching vibrational modes appear at 175 ± 2 cm^−1^, 245 ± 18 cm^−1^, 280 ± 2 cm^−1^ and 560 ± 30 cm^−1^ (See Figure S3 in SI), respectively, which are in agreement with the reported data^47^,^48^,^49^,^50^. As shown in Fig. 4 (inset), the interplanar distances of GNPs obtained from High-resolution TEM (HRTEM) characterizations are approximately 0.20 nm, which is the same as that of the {200} planes in crystalline bulk gold^51^. The surface charge of these GNPs was also studied by zeta potential measurements. The zeta potential distribution of GNPs reduced by proteins (Pep is taken as example) is shown in Figure S4 (SI) and the average value is about −26 eV. Zeta potential indicates negative surface properties of the GNPs with good stability in suspension. The green reducing reagents of proteins are expected to synthesize other noble metal nanoparticles (e.g. Ag NPs). The color and UV-vis absorption spectrum of Ag NPs reduced by Col in alkaline solution are shown in Figure S5 (SI), and the absorption peak appears at ~ 378 nm.

### Fabricating a multidimensional sensing platform

#### Qualitative detection of proteins

**Figure 5:**
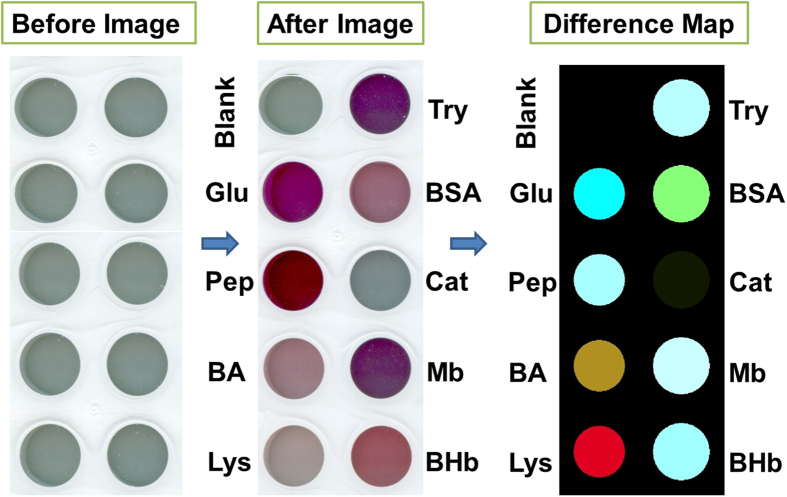
Color images of the Au(I) anions, luminol and H_2_O_2_ mixture in the absence (named “before” images) and presence (named “after” images) of proteins at 25 μg/mL. For display purpose, the color ranges of the difference maps are expanded from 5 to 8 bits per color (RGB range of 4–35 expanded to 0–255).

Based on the experimental phenomena that the distinctive color changes of GNPs reduced by different proteins in the presence of luminol and H_2_O_2_ are observed even by naked eye (Fig. 3a), a multidimensional sensing platform could be attempted to fabricate through simply extracting red, green and blue (RGB) alterations from the color responses in the process of reduction. For qualitative comparisons of these color changes, an ordinary flatbed scanner (or even a digital camera) is used to acquire digital images. As shown in Fig. 5, from the color profiles of the Au(I) anions, luminol and H_2_O_2_ mixture (as indicator) in the absence (named “before” images) and presence (named “after” images) of proteins, distinctive color changes are observed to each of the nine proteins. Taking the difference of the RGB values from the “before” and “after” images, the difference map is obtained. The enrichment-based colorimetric sensor array is successful at detecting and identifying these nine proteins at 25 μg/mL as demonstrated by the difference maps shown in Fig. 5. Even by eye, without statistical analysis, the array response to each protein is represented by a unique pattern. Excellent discrimination among these proteins is as well observed. The identification concentration (25 μg/mL) is competitive compared with the value (300 μg/mL) discriminated by other sensor array^30^.

**Figure 6:**
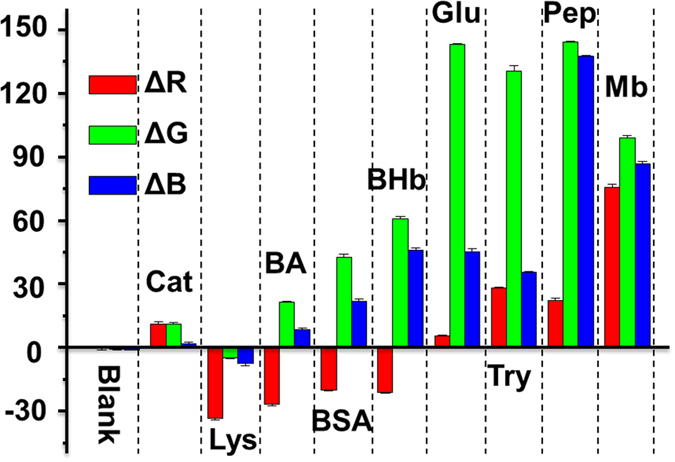
ΔRGB response patterns of the Au(I) anions, luminol and H_2_O_2_ mixture in the absence and presence of nine proteins (25 μg/mL). Each value is an average of five parallel measurements, and the error bars are shown.

**Figure 7:**
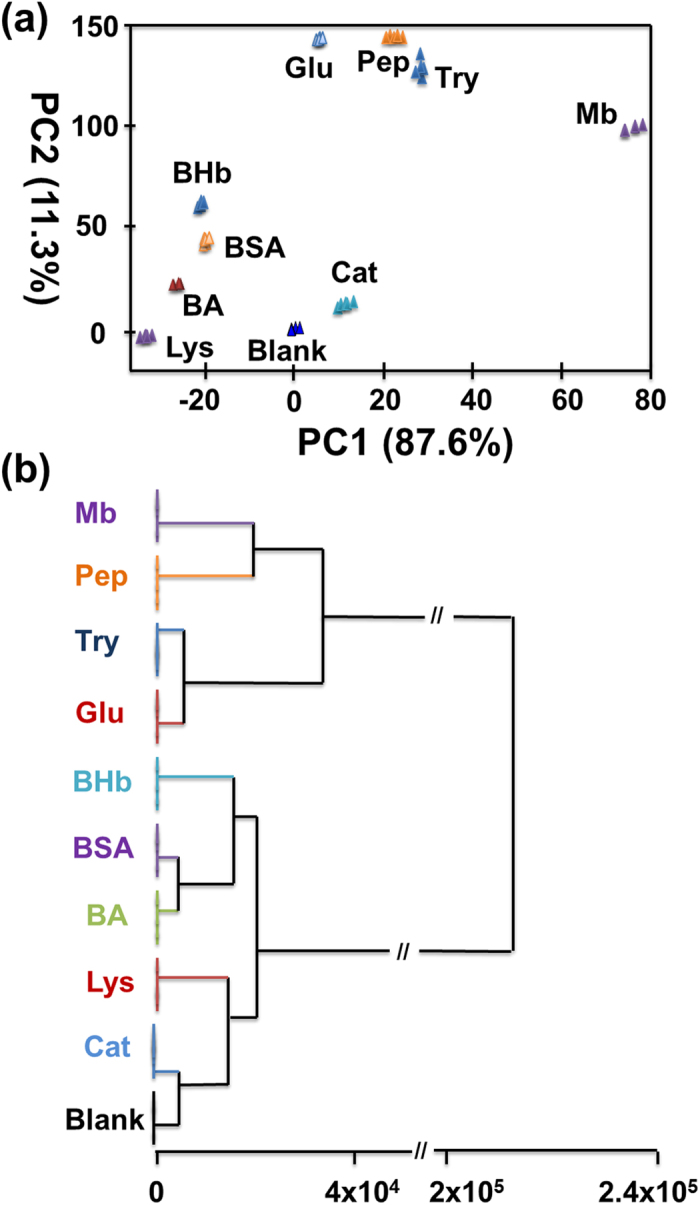
(**a**) Canonical score plots for the ΔRGB response pattern analyzed by PCA. (**b**) HCA analysis of the Au(I) anions, luminol and H_2_O_2_ mixture in the absence and presence of nine proteins (25 μg/mL) with five parallel measurements.

Quintuplicate data are acquired to investigate the reproducibility of the sensor array response to each protein. As shown in Fig. 6, the ΔRGB response patterns (can also be called fingerprints) induced by different proteins is distinct, suggesting the feasibility of protein indentification using such a sensor array. The RGB alterations of the Au(I) anions, luminol and H_2_O_2_ mixture in the absence and presence of nine proteins are listed in Table S1 (SI). The high dispersion of the enrichment-based sensor array data requires a classification algorithm that uses the full dimensionality of the data. Hierarchical clustering analysis (HCA) and principal component analysis (PCA) provide the simplest approaches^52^,^53^,^54^. As shown in Fig. 7a, the PCA reduced the size of the training matrix (1 indicator × 10 samples × 5 replicates) and transformed them into canonical factors. The canonical patterns are clustered into ten different groups, which are visualized as a well-clustered two-dimensional plot. The two factors are generated (87.6% and 11.3%) by PCA that are the combinations of the response patterns (3 channels × 10 samples × 5 replicates). The Au(I) anions, luminol and H_2_O_2_ mixture in the absence and presence of nine proteins at 25 μg/mL are analyzed using HCA, which is a statistical calssification method based on Euclidean distance (ED = [(ΔR)^2^ + (ΔG)^2^ + (ΔB)^2^]^1/2^). All of the 50 cases (10 samples × 5 replicates) are correctly assigned to their respective groups (Fig. 7b). Significantly, the distinct color response patterns are highly reproducible and characteristic for particular proteins, indicating the excellent reproducibility of protein identification. It’s worth mentioning that the different RGB responses are generated from the formation process of GNPs by different proteins.

#### Semiquantitative analysis of proteins

**Figure 8:**
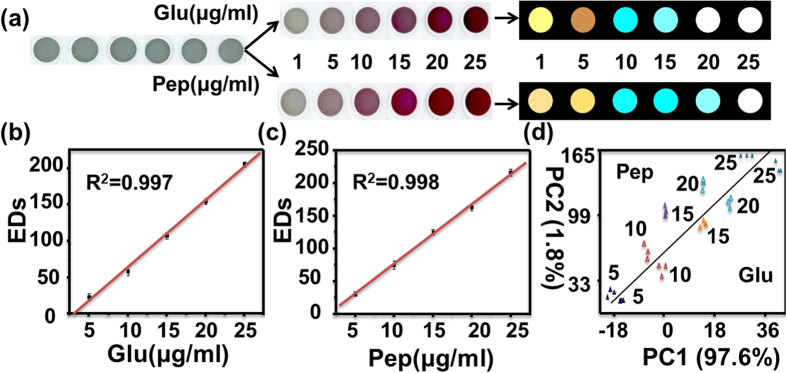
Performances of the as-developed sensing platform to individuals of proteins. (**a**) Color images of the Au(I) anions, luminol and H_2_O_2_ mixture before and after exposure to different concentrations of Glu and Pep, along with the color difference maps. For display purpose, the color ranges of the difference maps are expanded from 4 to 8 bits per color (RGB range of 4–19 expanded to 0–255). (**b**,**c**) The total EDs versus different concentrations of Glu and Pep, respectively. (**d**) PCA plot for the discrimination of Glu and Pep at different concentrations based on the RGB alterations of the as-developed system. The error bars in Fig. 8b,c represent the standard deviations of triplicate experiments.

For semiquantitative analysis of an individual protein, Glu and Pep are taken as examples. Their six concentration values ranging from 1 to 25 μg/mL were analyzed by using the as-developed multidimensional sensing platform. As shown in Fig. 8a, the color changes and difference maps both show that the more and more intense color responses of the as-developed indicator to the increasing concentrations of the two proteins. The total EDs were then applied to the concentration-dependent color response. The EDs versus different concentrations of the two representative proteins are shown in Fig. 8b,c. The linear relationship (R^2^ = 0.997 and 0.998) can be inferred, manifesting the dynamic ranges from 5 to 25 μg/mL and limits of detection (LODs) being 0.35 μg/mL and 0.24 μg/mL for Glu and Pep, respectively (Table S2, SI). To reveal the nature of the linear colour degree of GNPs associated with the detected protein concentration (Pep is taken as example), we characterized the UV-vis absorption spectra of GNPs generated in the reduction process. Figure S6 (SI) shows the absorption spectra of the Au(I) anions, luminol and H_2_O_2_ mixture in presence of different concentrations of Pep. The absorption peak is clearly blue-shifted and its intensity increases with the increase of Pep concentration. According to Mie theory^45^, the position and intensity variations of the UV-vis absorption bands indicate that the particle size of generated GNPs gradually decreases and their corresponding concentration increases with the increasing of Pep concentration. Interestingly, although the as-synthesized GNPs solution reduced by the same concentrations of Glu and Pep display the similar colors, the resulting two-dimensional PCA score plot (Fig. 8d) yields a clear separation of same and even different concentrations of Glu and Pep. Therefore, the as-developed sensing platform based on *in-situ* reduction method to synthesize GNPs of different sizes demonstrates not only great potentials in detection of multiple proteins, but qualitative and even semiquantitative differentiation of individuals.

#### Applicability for real samples

The performance of the triple-channel sensing platform was demonstrated for the discrimination of proteins in the presence of human urine. Here, the human urine was firstly spiked with the nine proteins, and then subject to the same analysis process as that of in deionized water. The final concentration of proteins contained in the mixture is 25 μg/mL. As shown in Figure S7 (SI), even by the naked eye, discinct color response profiles of the as-developed indicator in the absence and presence of nine proteins are observed both in the digital photo and difference map. The triple-channel sensing platform exhibits distinct ΔRGB recognition patterns (fingerprints, Figure S8, SI), which are acquired by taking quintuplicate experiments and probe reproducibility of the sensing system. To expose the fingerprints more clearly, the triple-channel response patterns are subjected to PCA (3 channels × 10 samples × 5 replicates), which demonstrates that the canonical response patterns of the as-developed indicator in the absence and presence of nine proteins are clearly clustered into ten groups (Figure S9, SI). Moreover, the as-developed indicator in the absence and presence of nine proteins are accurately classified with no errors out of 50 cases (Figure S10, SI). Although human urine with more than 1500 different proteins^55^ generates a complex matrix that is challenging for the as-developed sensing platform, each of the spiked proteins in the urine sample still generates distinct responses.

After successful discrimination of the spiked proteins in human urine, the next challenge is to identify proteins at unknown concentrations in complex matrix (e.g. human tear). The as-developed sensing platform was further demonstrated for the identification of Lys in human tear. As shown in Figure S11 (SI inset), comparing with the color responses of the as-developed indicator to the concentrations of Lys contained in water, the concentration of Lys in human tear should be in the range of 0.72–1.1 mg/ml (equal to (9–14) μg/mL × 80.5). The EDs versus different concentrations of Lys in water is shown in Figure S11 (SI). Based on the corresponding linear relationship (R^2^ = 0.994), and the ED data (36.09 ± 2.25) obtained from the human tear, we find that the concentration of Lys in human tear is 0.89 ± 0.11 mg/mL (See Table 1 and the calculation method in SI), which is in good agreement with the reported value^56^. Therefore, the as-developed sensing platform demonstrates great potentials in the qualitative and semiquantitative determination of target proteins in complex matrix.

## Discussion

In summary, we develop an *in-situ* reduction-based method to synthesize highly monodispersed, spherical GNPs by using the reducing agents of proteins. The protein of Col turns Au(III) complexes into the aqueous Au(I) ainions, which further reduced by other proteins to spherical GNPs of different sizes. The UV-vis absorption spectra of GNPs are found to be characteristic for a given protein. And the absorption wavelength values and sizes of spherical GNPs are ranged from 530 to 600 nm and from 40 to 110 nm, respectively. Interestly, the prepared GNPs are found to be with the exposed {100} facets and absorbed by proteins via Au-O, Au-N, Au-S and Au-C interactions.

Through simply extracting the color responses, such as RGB alterations, we achieve the discrimination of proteins in the process of preparing GNPs. We have observed the different recognition patterns (fingerprints), and HCA/PCA further demonstrate its detection and discrimination capability. This as-developed *in-situ* reduction-based sensing platform could as well as be applied in qualitative and semiquantitative analysis of a specific protein with high sensitivity based on their corresponding color response profiles and EDs fitting curves, respectively. Moreover, this multidimensional sensing platform also offers great potential for the discrimination of proteins in real samples (e.g. urine and tear).

## Methods

### Materials and instruments

Collagen (Col), myoglobin (Mb), glucoamylase (Glu), catalse (Cat) were from Aladdin Reagent Co. Ltd (Shanghai, China). Bovine serum albumin (BSA) was purchased from Gibco (Grand Island, USA). Lysozyme (Lys), pepsin (Pep), trypsin (Try), bovine albumin (BA), bovine hemoglobin (BHb), chloroauric acid tetrahydrate (HAuCl_4_•4H_2_O), NaOH, NaH_2_PO_4_·2H_2_O, Na_2_HPO_4_·12H_2_O, NaCl, KCl and CaCl_2_ were obtained from Sinopharm Chemical Reagent Co., Ltd (Beijing, China). All chemicals were used as received without further purification. 96-well plates (Corning 3632) were obtained from Genetimes Technology. The stock solutions of proteins were prepared using 6 mM phosphate buffered saline (PBS, pH 7.4) buffer.

The pH measurements were performed using a PHS-3C pH meter. For all sensing experiments, imagings were acquired with a flatbed scanner (Epson Perfection V300) in 96-well plates. Transmission electron microscopy (TEM) was performed on a Tencai F20 instrument and operated at 200 kV. UV-vis absorption spectra were recorded using a Lambda 950 UV-vis spectrophotometer from Perkin Elmer. Zeta-potential of GNPs was measured using a Microtrac S3500 (Microtrac Inc., USA), and measured at 25 °C. FT-IR spectroscopy was performed using a Nicolet 6700 spectrometer.

### Preparation of Au(I) anions

20 μL Col (1 mg/mL) was added to 6 mL HAuCl_4_ (1 mM), and then added 50 μL NaOH solution (4M, pH = 13.0), the developed colorless Au(I) anions was prepared and stored at 4 °C until use.

### GNPs preparation

8 μL of Luminol (10 mM, dissolved in 0.2 M NaOH solution), 100 μL of H_2_O_2_ (1 M) and 15 μL of proteins (4 mg/mL, dissolving in deionized water) was firstly added to 585 μL PBS (6 mM, pH = 7.4), and then mixed with 500 μL Au(I) anions, the mixture was incubated at 37 °C for 30 min.

### Proteins detection and discrimination

Different concentrations of proteins (15 μL) were added into the mixture of PBS (585 μL, 6 mM), Luminol (8 μL, 10 mM), H_2_O_2_ (100 μL, 1 M) and Au(I) solution (500 μL), then incubated at 37 °C for 30 min.

300 μL of the control (without proteins) and work solutions (in the presence of proteins) were loaded into a 96-well plate, respectively, and the “before” (from the control solutions) and “after” (from the work solutions) images were acquired on an Epson Perfection V300 photo flatbed scanner. Difference maps were acquired by taking the difference of the RGB values from the center of the indicator solution (in 96-well plates) from the “before” and “after” images using the commercial Photoshop software.

The chemometric analysis was performed on the color difference vectors using the Multi-variate statistical package (MVSP v.3.1, kovach computing); in all cases, hierarchical cluster analysis (HCA) and principal component analysis (PCA) were performed based on the database library (Tables S1 and S3, SI) using the minimum variance for classification.

### Analysis of proteins in human urine and tear

Proteins detection in human urine and tear was taken as an example to preliminarily test the capability of the as-developed sensing platform for real samples. The tear and first morning urine samples were collected from healthy volunteers in Nanyang People’s Hospital, China. The spiked samples were obtained by adding proteins to the dialysates of human urine. The final concentration of the target proteins is 25 μg/mL. The analysis procedure of proteins in human urine is similar with that in deionized water.

The developed method was further used for the determination of target proteins in complex matrix (e.g. Lys in human tear). Different concentrations of Lys contained in water (15 μL) and 15 μL human tear were added into the mixture of PBS (585 μL, 6 mM), Luminol (8 μL, 10 mM), H_2_O_2_ (100 μL, 1 M) and Au(I) solution (500 μL), then incubated at 37 °C for 30 min, respectively. The Lys concentration was diluted 80.5 times in the analytical process and the final concentration of the target Lys in water is 1 μg/mL, 9 μg/mL, 14 μg/mL, 22 μg/mL and 35 μg/mL, respectively. All the experiments were performed in accordance with Nanyang People’s Hospital Ethics Committee’s guidelines and regulations.

### Ethics statement

All experiments and procedures were performed in accordance with the appropriate guidelines. All procedures were approved by Nanyang Normal University. Informed consent was obtained from all volunteers before being enrolled in the study.

## Additional Information

**How to cite this article**: Leng, Y. *et al*. Protein-directed synthesis of highly monodispersed, spherical gold nanoparticles and their applications in multidimensional sensing. *Sci. Rep.* **6**, 28900; doi: 10.1038/srep28900 (2016).

## Supplementary Material

Supplementary Information

## Figures and Tables

**Figure 1:**
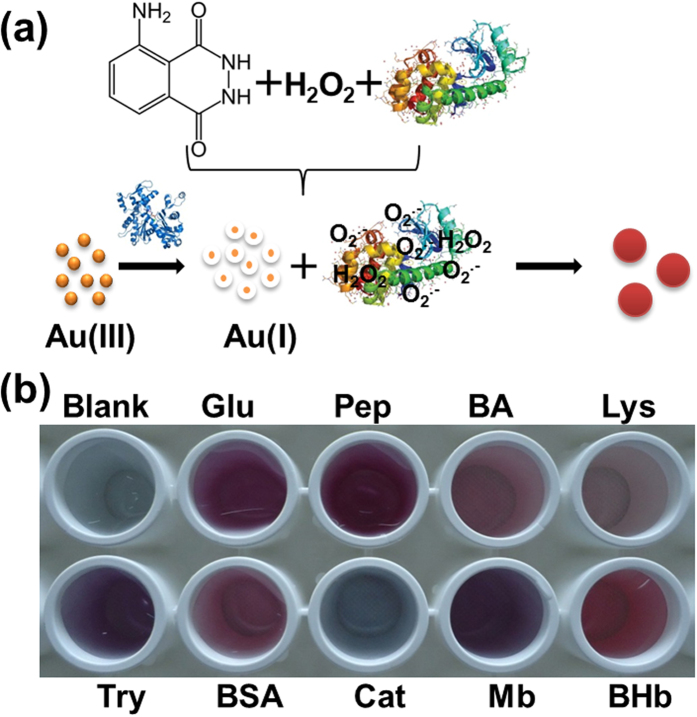
(**a**) Schematic illustration of an *in-situ* reduction method to synthesize monodispersed and spherical GNPs by using proteins, the reducing power of which activated by H_2_O_2_ and the superoxide anion (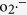). (**b**) The distinctive colors of as-synthesized GNPs reduced by different proteins.

**Figure 2:**
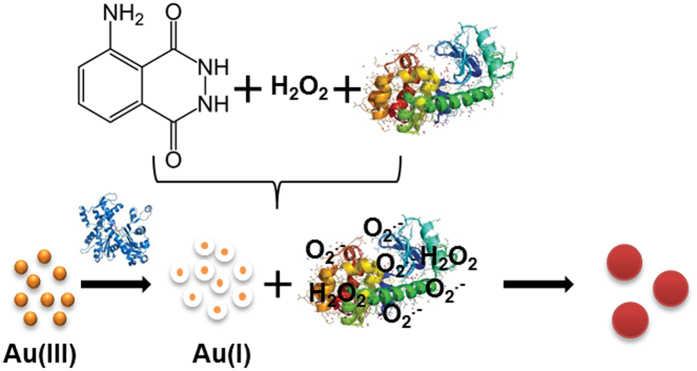
Schematic illustration of an *in-situ* reduction method to synthesize monodispersed and spherical GNPs by using proteins, the reducing power of which activated by H_2_O_2_ and the superoxide anion 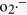.

**Table 1:** Determination of Lys in human tear using the developed procedure.

Sample	[Lys] (mg/mL)	
	Found*	Ref. 56
Human tear	0.89 ± 0.11	0.72–1.10

^*^mean ± standard deviation, n = 3.
