# Supplementary material for: Protein-directed synthesis of highly monodispersed, spherical gold nanoparticles and their applications in multidimensional sensing
Source: Sci Rep. 2016 Jun 29;6:28900. doi: 10.1038/srep28900 (PMC4926111; doi:10.1038/srep28900)
Supplement: Supplementary Information [file srep28900-s1.doc]

Supplementary Information

Protein-directed synthesis of highly monodispersed, spherical gold nanoparticles and their applications in multidimensional sensing

Yumin Leng^1^, Ling Fu^3^, Liqun Ye^2^, Bo Li^2^, Xiumei Xu^1^, Xiaojing Xing^2^, Junbao He^1^, Yuling Song^1^, Chaoliang Leng^3^, Yongming Guo^2^, Xiaoxu Ji^1^ & Zhiwen Lu^1^

^1^College of Physics and Electronic Engineering, Nanyang Normal University, Nanyang 473061, China

^2^College of Chemistry and Pharmaceutical Engineering, Nanyang Normal University, Nanyang 473061, China

^3^College of Agricultural Engineering, Nanyang Normal University, Nanyang 473061, China

**Experimental Methods**

**Preparation of Silver Nanoparticles.** 20 μL Col (1 mg/mL) was added to 6 mL AgNO_3_ (1 mM), and then added 100 μL NaOH solution (1M), the silver nanoparticles were prepared.

**Supplementary Figures**


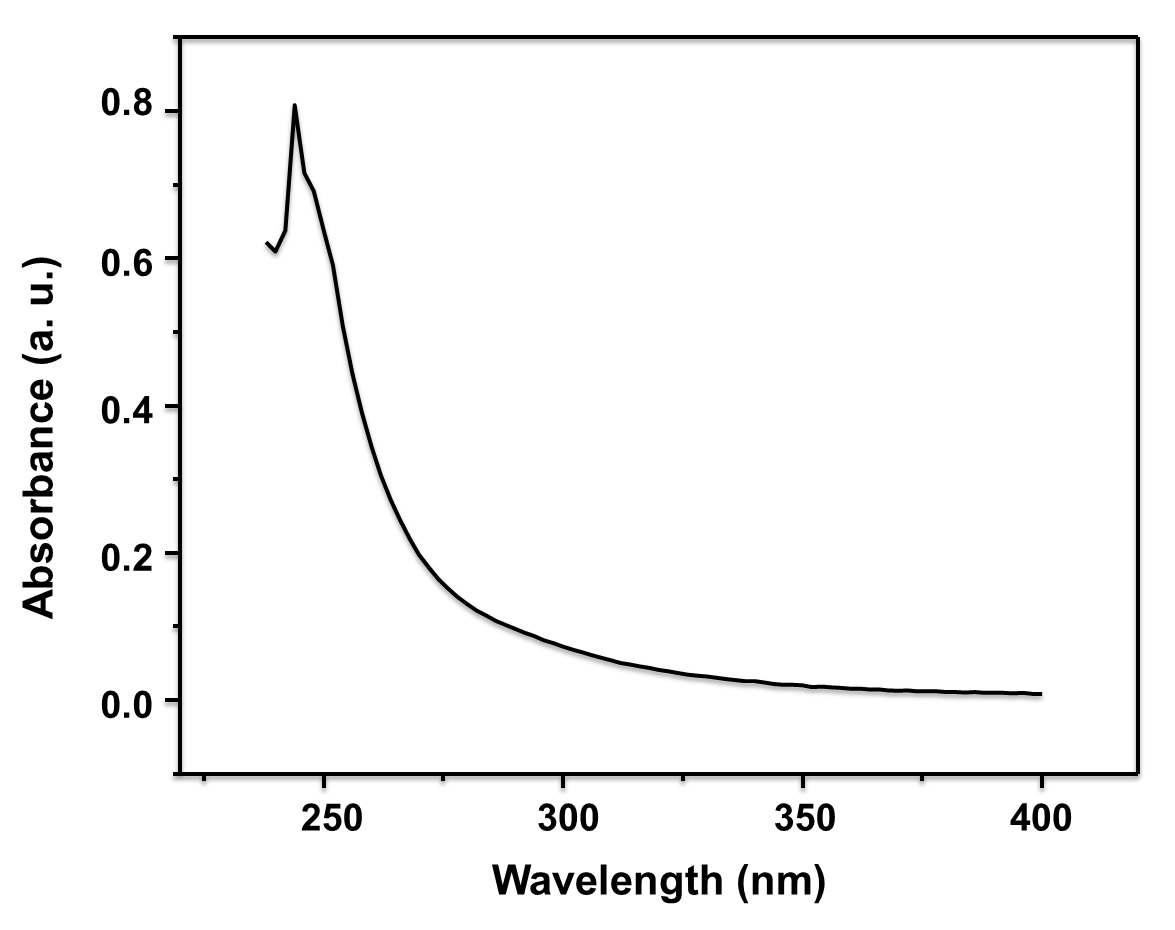


**Figure S1.** UV-vis absorption spectra of the aqueous Au(I) anions.

**Figure S2.** Digital photos of the aqueous Au(I) anions before and after exposure to 25 μg/ml proteins.


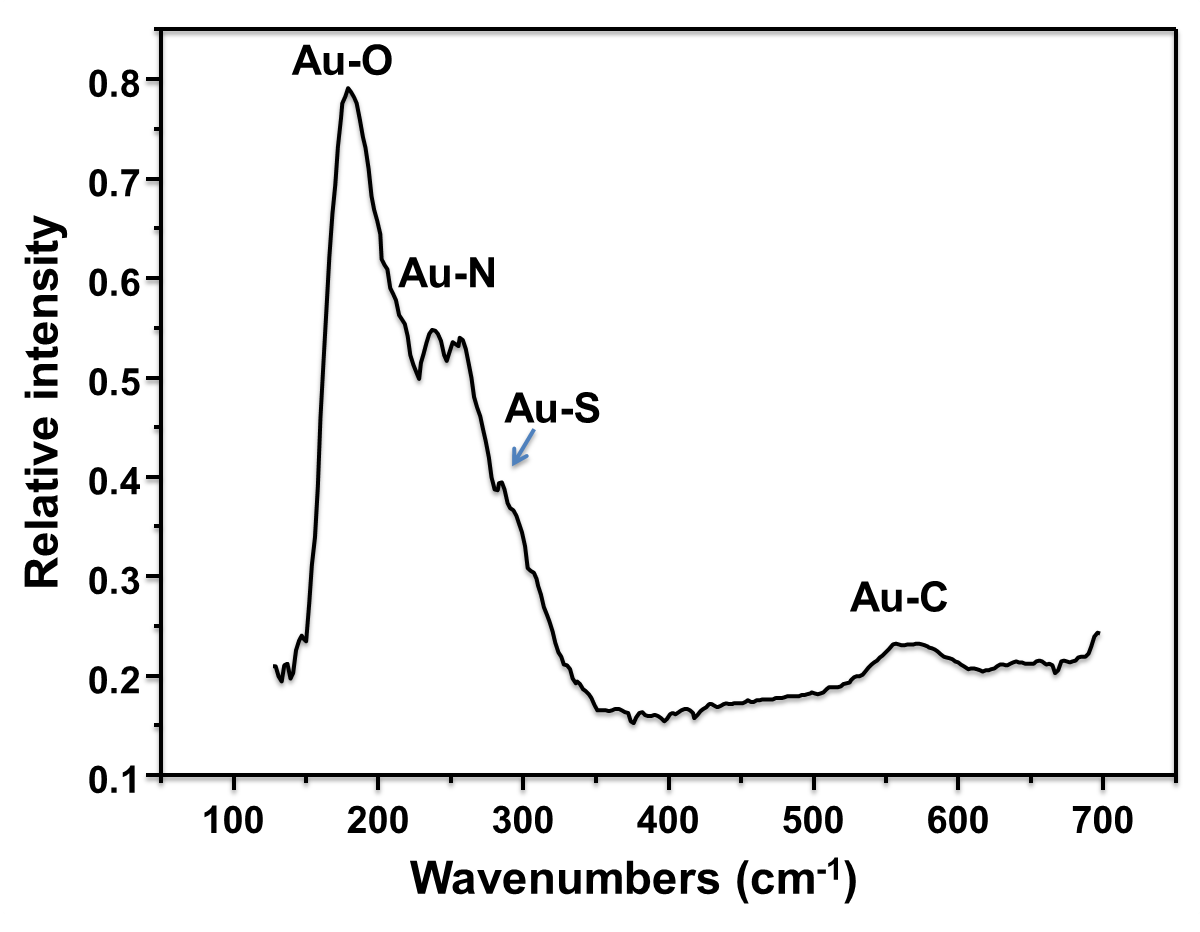


**Figure S3.** The FT-IR spectrum of GNPs coated by proteins (e.g. Pep).


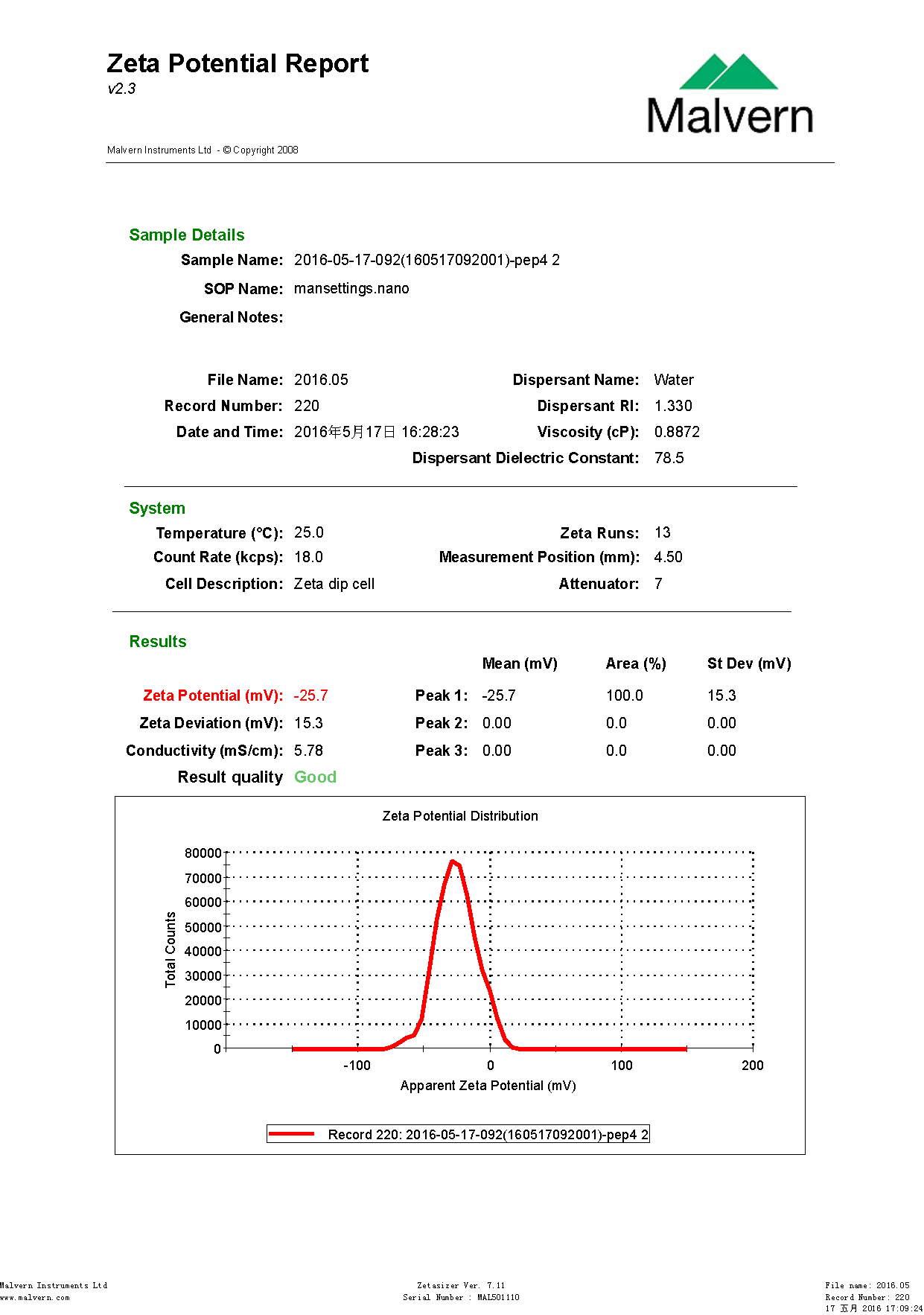


**Figure S4.** The zeta potential distribution of GNPs reduced by proteins (e.g. Pep).


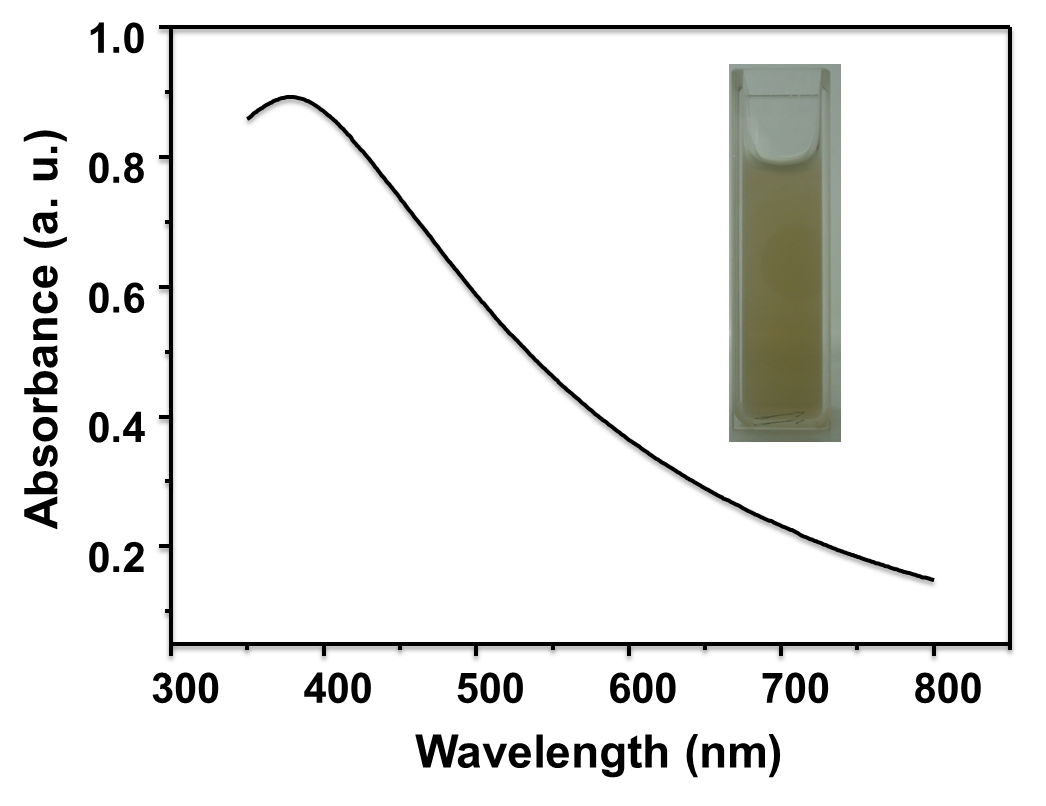


**Figure S5.** UV-vis absorption spectrum of Ag NPs reduced by Col in alkaline solution. Inset: digital photo of Ag NPs.


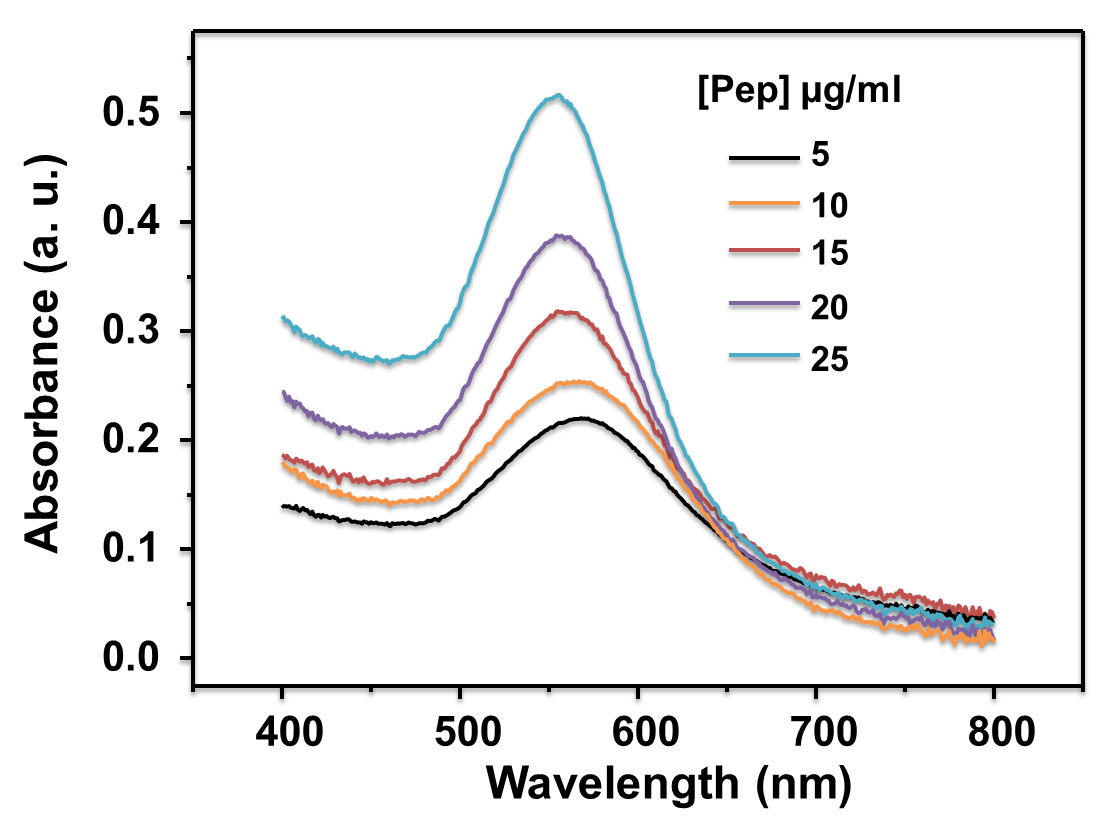


**Figure S6.** The UV-vis absorption spectra of the Au(I) anions, luminol and H_2_O_2_ mixture in the presence of different concentrations of Pep.

**Figure S7.** Color images of the as-developed indicator before and after exposure to the spiked proteins in human urine, and the color difference map. With display purposes, the color range of the difference map is expanded from 5 to 8 bits per color (RGB range of 4−35 expanded to 0−255).


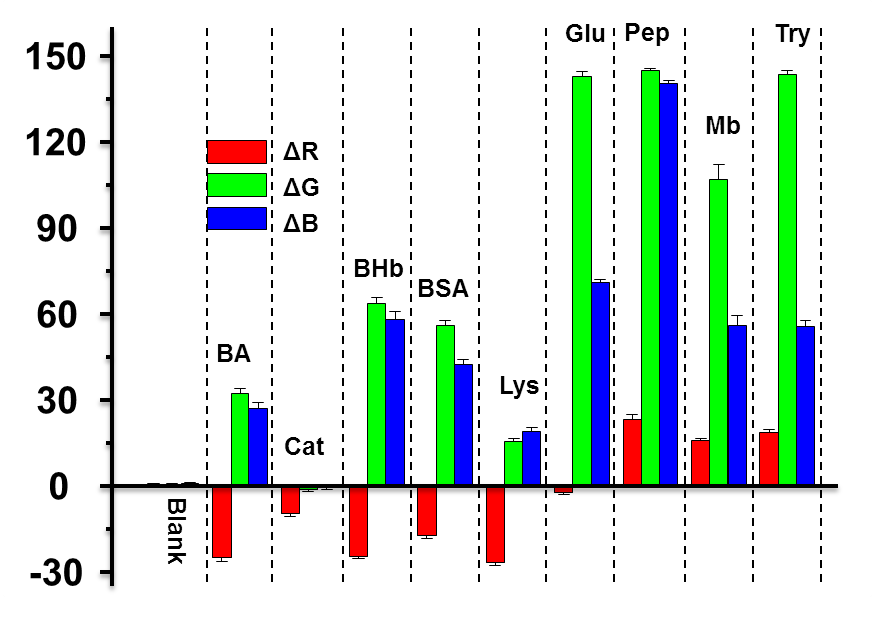


**Figure S8.** Recognition patterns for the spiked proteins in human urine and a control based on their corresponding ΔRGB values obtained from the “before” and “after” images (See Figure S7). Error bars represent standard deviations of five parallel measurements.


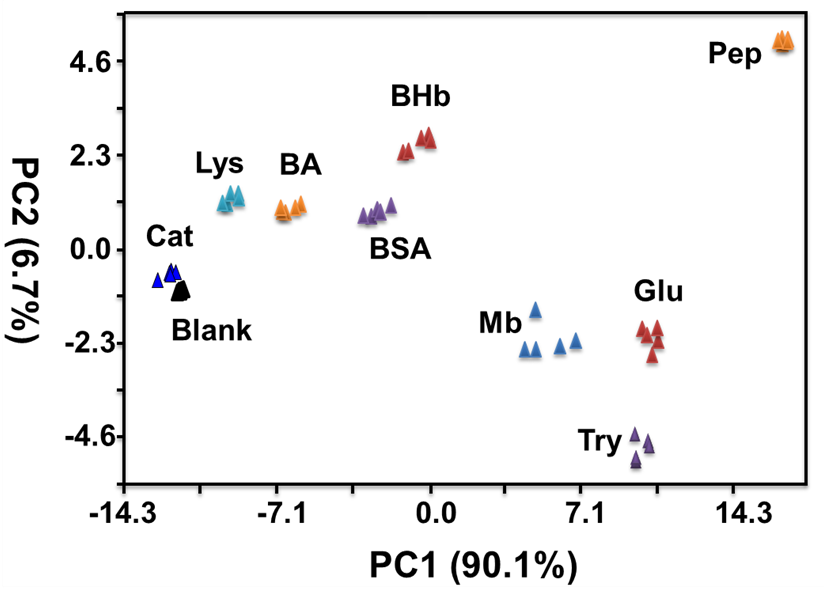


**Figure S9.** PCA for the discrimination of the spiked nine proteins in human urine and a control based on the triple-channel properties of as-developed sensor array.


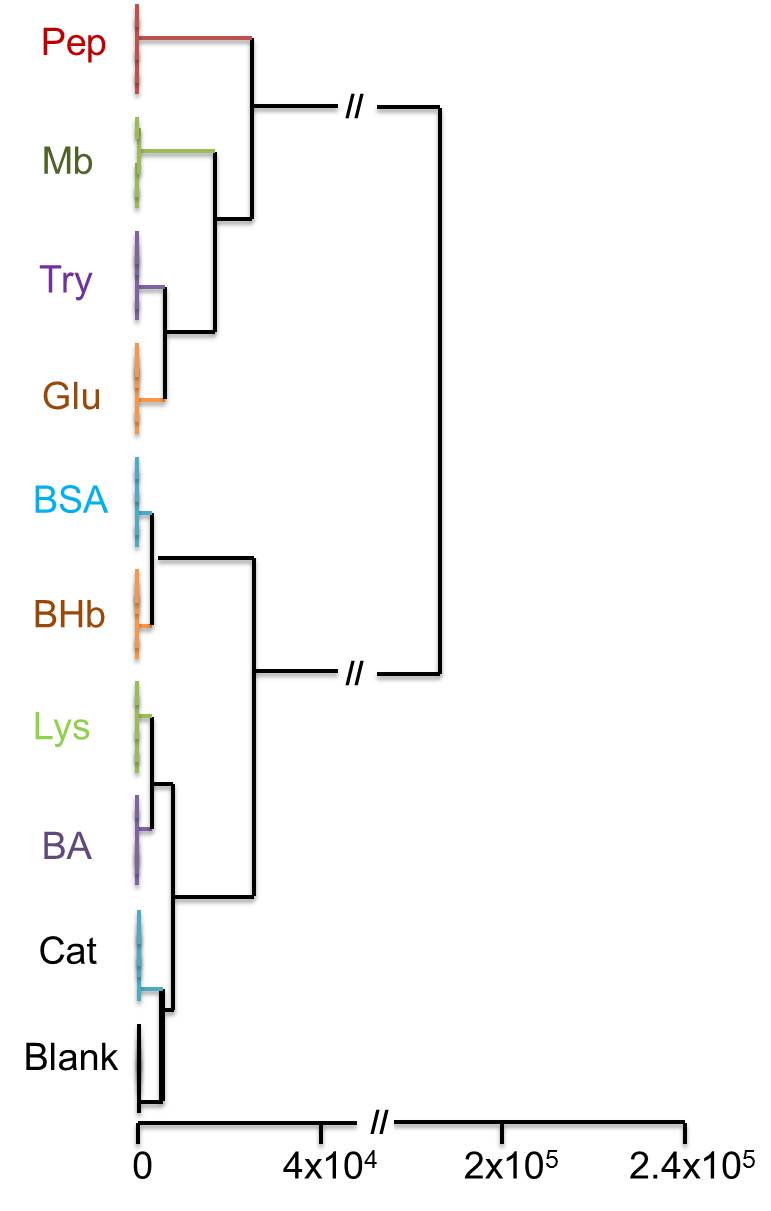


**Figure S10.** HCA for the discrimination of the spiked nine proteins in human urine and a control based on the as-developed sensor array. No confusions or errors in classification were observed in 50 trials.


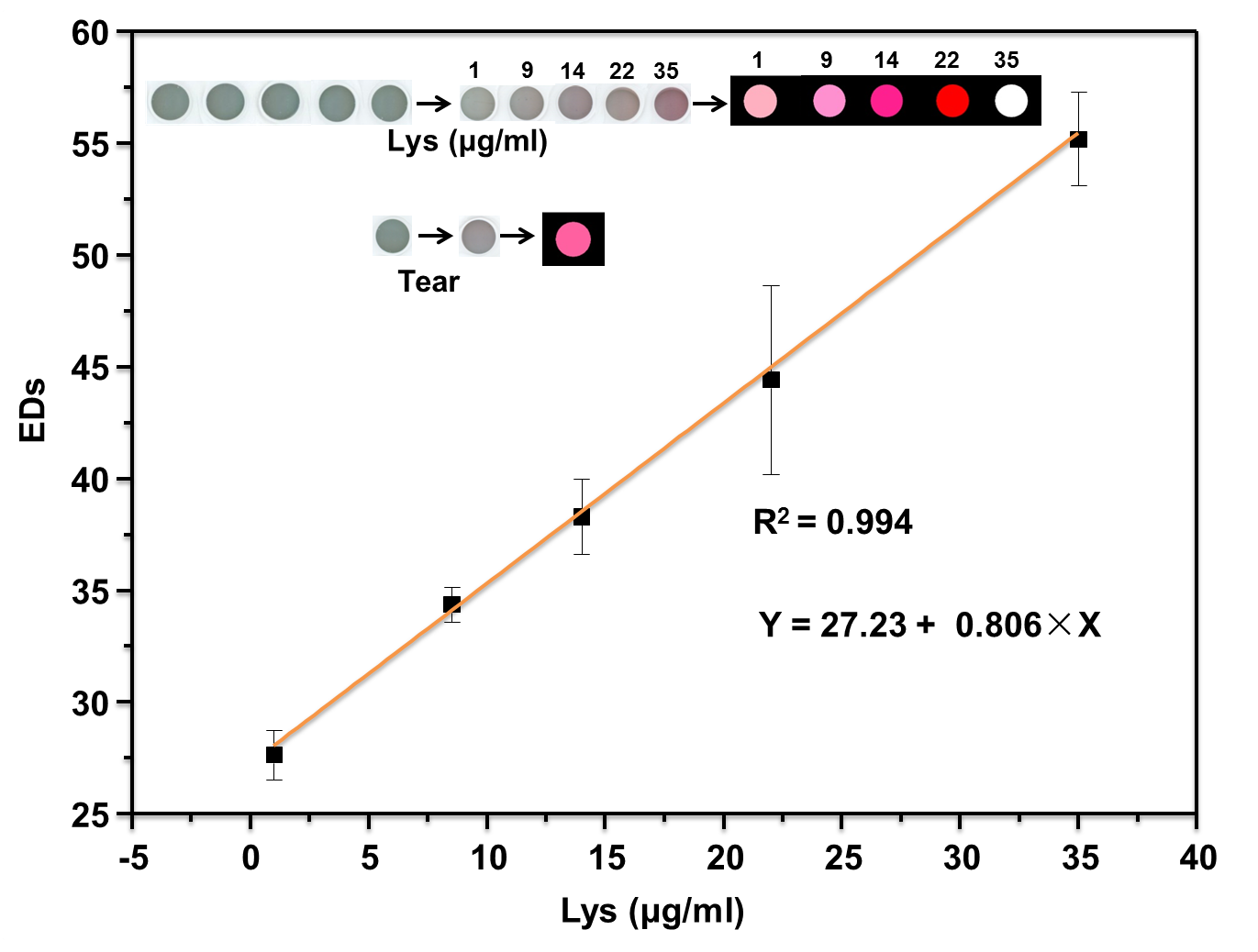


**Figure S11.** **Performances of the as-developed sensing platform to Lys in water and tear**. The total EDs versus different concentrations of Lys contained in water. Inset: Color images of the as-developed indicator before and after exposure to different concentrations of Lys in water and tear, along with the color difference maps. The calculation method for the concentration of Lys contained in human tear: Y_ED of tear_ = 36.09 ± 2.25 = 27.23 + 0.806×X → X = 11 ± 1.4 µg/ml → The concentration of Lys in human tear = (11 ± 1.4)×80.5 µg/ml = 0.89 ± 0.11 mg/ml. Note: The Lys concentration is diluted 80.5 times in the analytical process.

**Supplementary Tables**

**Table S1.** Database of the RGB alterations of the Au(I) anions, luminol and H_2_O_2_ mixture in the absence and presence of nine proteins (25 μg/ml, dissolving in deionized water).

|  | R | G | B |  | R | G | B |
| --- | --- | --- | --- | --- | --- | --- | --- |
| Blank-1  Blank-2  Blank-3  Blank-4  Blank-5  BA-1  BA-2  BA-3  BA-4  BA-5  Cat-1  Cat-2  Cat-3  Cat-4  Cat-5  BHb-1  BHb-2  BHb-3  BHb-4  BHb-5  BSA-1  BSA-2  BSA-3  BSA-4  BSA-5 | 0.114  1.048  -0.785  -0.761  -0.884  -25.706  -27.263  -26.212  -27.517  -25.961  9.625  10.386  11.475  11.812  13.165  -21.009  -21.804  -20.985  -21.278  -20.483  -19.761  -20.556  -20.338  -20.031  -19.236 | -0.277  -0.275  -0.766  -1.027  -1.455  21.619  21.165  22.159  21.176  21.630  9.232  11.056  11.576  11.446  12.559  60.357  59.387  62.341  60.969  61.939  41.679  40.709  43.844  43.409  44.380 | -0.063  -0.663  -0.774  -1.176  -1.582  8.724  9.667  9.623  8.410  7.468  0.053  2.177  2.101  2.600  2.722  45.284  44.098  47.377  46.429  47.615  21.084  19.898  22.365  22.432  23.618 | Lys-1  Lys-2  Lys-3  Lys-4  Lys-5  Glu-1  Glu-2  Glu-3  Glu-4  Glu-5  Pep-1  Pep-2  Pep-3  Pep-4  Pep-5  Mb-1  Mb-2  Mb-3  Mb-4  Mb-5  Try-1  Try-2  Try-3  Try-4  Try-5 | -32.050  -33.607  -33.500  -34.760  -33.204  5.674  4.879  5.554  5.491  6.286  21.387  20.592  24.262  22.252  23.047  74.104  74.255  76.458  76.500  78.190  28.545  28.211  28.977  28.349  27.137 | -4.299  -4.753  -4.369  -5.295  -4.841  143.792  142.822  143.446  143.024  143.994  144.822  143.851  144.059  143.825  144.796  97.833  97.839  99.612  99.710  100.693  123.915  135.873  128.567  129.406  126.757 | -6.945  -6.003  -7.042  -8.153  -9.096  44.182  42.996  46.043  46.505  47.690  138.450  137.264  137.735  136.673  137.859  85.063  85.139  87.617  87.603  88.224  36.091  35.458  35.919  36.087  35.185 |

**Table S2.** The equation and parameters for limit of detection (LOD) calculation.

| Y = A + S × X | | | | | | |
| --- | --- | --- | --- | --- | --- | --- |
| Parameter | A | S | R | SD | N | P |
| Glu | -28.71637 | 9.2566 | 0.99863 | 1.09019 | 5 | <0.0001 |
| Pep | -15.08698 | 9.13219 | 0.99918 | 0.733695 | 5 | <0.0001 |

A = Y-intercept, S = slope, R = correlation, SD = standard deviation, N = number of data points, P = probability value.

The limit of detection (LOD) could be obtained by the above equation and parameters.

LOD = 3×SD/S

LOD_Glu_ = 3×1.09019/9.2566 = 0.35 μg/ml

LOD_Pep_ = 3×0.733695/9.13219 = 0.24 μg/ml

**Table S3.** Database of the RGB alterations of the Au(I) anions, luminol and H_2_O_2_ mixture in the absence and presence of nine proteins (25 μg/ml, dissolving in Urine).

|  | R | G | B |  | R | G | B |
| --- | --- | --- | --- | --- | --- | --- | --- |
| Blank-1  Blank-2  Blank-3  Blank-4  Blank-5  BA-1  BA-2  BA-3  BA-4  BA-5  Cat-1  Cat-2  Cat-3  Cat-4  Cat-5  BHb-1  BHb-2  BHb-3  BHb-4  BHb-5  BSA-1  BSA-2  BSA-3  BSA-4  BSA-5 | 0.114  1.048  0.785  0.761  0.884  -24.904  -22.838  -26.428  -22.998  -26.355  -11.721  -9.771  -9.758  -7.546  -8.627  -25.285  -23.812  -24.944  -22.694  -24.528  -17.593  -15.221  -18.193  -16.229  -18.456 | 0.277  0.275  0.766  1.027  1.455  32.003  35.059  31.416  33.889  30.658  -2.945  -0.657  -0.851  0.201  -0.761  60.587  65.888  64.414  66.632  61.823  55.045  59.032  56.309  56.885  52.908 | 0.063  0.663  0.774  1.176  1.582  26.194  30.201  25.488  28.760  25.916  -3.045  0.842  0.203  1.718  0.268  54.369  61.405  59.469  61.079  55.551  40.780  46.014  42.734  43.095  39.376 | Lys-1  Lys-2  Lys-3  Lys-4  Lys-5  Glu-1  Glu-2  Glu-3  Glu-4  Glu-5  Pep-1  Pep-2  Pep-3  Pep-4  Pep-5  Mb-1  Mb-2  Mb-3  Mb-4  Mb-5  Try-1  Try-2  Try-3  Try-4  Try-5 | -27.273  -25.448  -27.786  -25.298  -27.764  -3.751  -0.778  -2.179  -1.374  -2.562  21.486  24.873  23.407  25.991  21.203  14.550  16.495  17.204  16.206  15.504  17.723  19.291  17.792  20.621  18.795 | 15.023  16.763  15.191  17.255  13.790  140.189  144.912  143.894  144.786  141.698  145.792  145.886  144.503  145.554  144.269  102.134  104.678  111.229  115.571  101.118  143.861  144.977  143.527  145.449  140.674 | 17.554  21.141  19.518  20.544  16.794  70.548  71.888  73.396  68.671  70.561  139.612  141.747  140.751  141.543  139.360  50.917  52.986  57.565  60.759  58.654  52.976  58.205  53.529  57.872  57.002 |
